# The Elusive Sarcoidosis, an Eight-Year Journey to the Diagnosis of Sarcoidosis: A Case Report

**DOI:** 10.7759/cureus.39400

**Published:** 2023-05-23

**Authors:** Alexandra Vacaru, Jasmine P Nguyen, Stacy J Youn, Donna Lien

**Affiliations:** 1 School of Medicine, Loma Linda University, Loma Linda, USA; 2 Internal Medicine, Loma Linda University Medical Center, Loma Linda, USA; 3 Anesthesiology/Internal Medicine, Loma Linda University Medical Center, Loma Linda, USA

**Keywords:** lymph nodes pathology, fna biopsy, fine-needle aspiration, antibiotics, adalimumab (humira), corticosteroids, risk of malignancy, diffuse lymphadenopathy, non-caseating granulomas, chronic sarcoidosis

## Abstract

We present a unique case of a patient coming to our internal medicine clinic with intermittent diffuse lymphadenopathy and non-specific symptoms for the past eight years. Initially, the patient was thought to have carcinoma of unknown primary origin, given the abnormalities seen in her imaging. The diagnosis of sarcoidosis was also dismissed, given that the patient had not responded to steroids with negative laboratory support. The patient was referred to several specialists, and only after a pulmonary biopsy was a non-caseating granuloma revealed after multiple prior failed biopsies. The patient was placed on infusion therapy and responded positively. This case demonstrates a challenging diagnosis and treatment which emphasizes the importance of considering alternative treatments if the initial therapy fails.

## Introduction

Sarcoidosis is an inflammatory multisystemic disease characterized by the formation of non-caseating granulomas resulting in non-specific clinical manifestations such as fatigue, myalgia, and erythema nodosum, commonly affecting the lungs, eyes, skin, liver, and lymph nodes; confirmation of the diagnosis often requires a histopathological examination [1,2]. The diagnosis is one of exclusion; sometimes, a biopsy may reveal a non-caseating granuloma, but the absence of it does not exclude the diagnosis [1]. It can mimic other disease processes because of its non-specific nature, and the condition may go undiagnosed for years. We present a challenging case of sarcoidosis, necessitating a collaborative approach from multiple medical specialties, extensive workup, and multiple trials of treatment.

## Case presentation

This case describes a 59-year-old Hispanic female presenting with chronic, fluctuating diffuse lymphadenopathy (LAD), escaping sarcoidosis diagnosis for eight years despite substantial laboratory blood work, extensive imaging, various procedures, and numerous biopsies. She has a history of hypertension, hypothyroidism, diabetes mellitus, inflammatory arthritis, and uterine cancer in remission with symptoms of night sweats, myalgia, fatigue, fever, abdominal pain, shortness of breath, and headaches. She also reported an unclear history of gastric lymphoma from an esophagogastroduodenoscopy (EGD) six years prior; however, the diagnosis was never confirmed pathologically, and the patient denied receiving any chemoradiotherapy. She has a family history of hypertension and unspecified cancer without any known work exposure to toxins or heavy metals.

**Figure 1:**
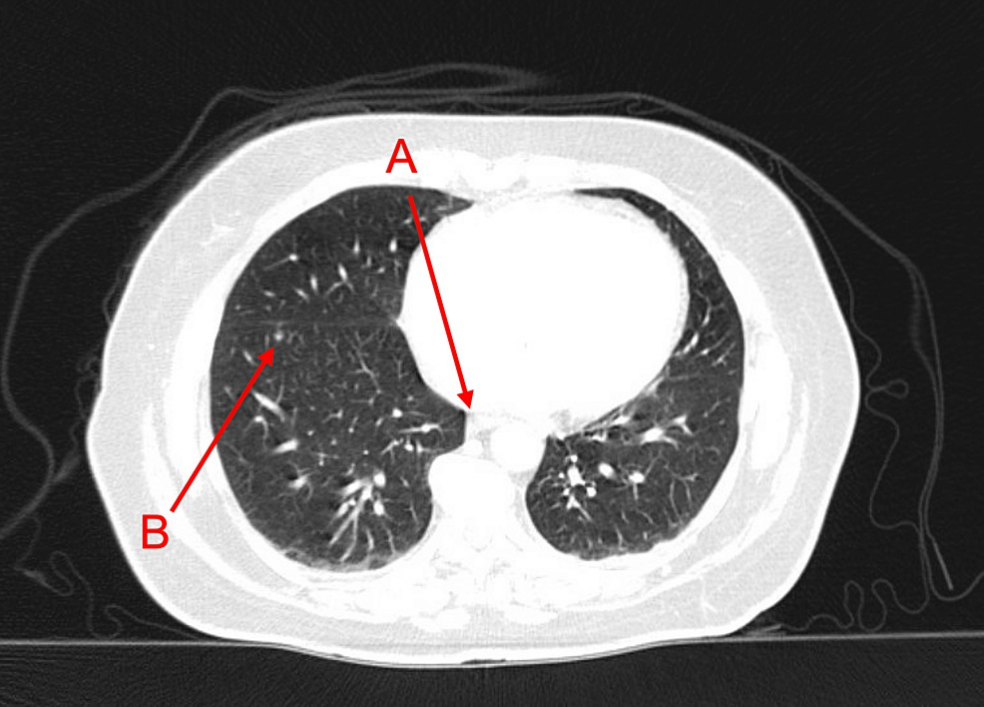
Computed tomography of the chest with contrast Computed tomography of the chest with contrast showing prominent mediastinal hilar lymph nodes (arrow A) and a right lower lobe pulmonary nodule measuring 4-5 mm (arrow B).

Over a seven-month period, she failed multiple antibiotic treatments prompting magnetic resonance imaging (MRI) for left submandibular swelling which showed enlarged lymph nodes and chronic sialadenitis in that region. Computed tomography (CT) and positron emission tomography (PET) scans additionally revealed LAD of the head, neck, chest, abdomen, and gluteus region measuring up to 1.1 cm with a standardized uptake value maximum of 3.3 notably in the hilar lymph nodes stimulating concern for malignancy and referrals to hematology-oncology, general surgery, and pulmonology (Figure 1). Infectious disease, malignancy, and other workup were negative (Table 1). Fine-needle aspiration (FNA) of the submandibular gland also showed non-specific granulomatous changes. Interventional radiology could not biopsy gastric lymph nodes due to their small size and other biopsies were unremarkable, including the mandibular gland and gluteal, inguinal, and pulmonary lymph nodes. Further rheumatologic workup was negative, and she was empirically trialed on six-week prednisone taper starting at 15 mg daily for a not yet diagnosed autoimmune process. Over the next years, her diagnostic medical journey continued.

**Table 1:** Autoimmune, infectious, malignancy, and other workup Autoimmune workup was negative, except for elevated Beta Globulin and Antinuclear Antibody With HEp-2 Substrate which was thought to be non-specific. Immunoglobulin G, Subclass 1 was elevated and thought to be related to her inflammatory arthritis. Infectious workup was negative. Malignancy workup was negative. Lead metal poisoning workup was negative, and calcium was normal.

Autoimmune Workup	
Lab Test	Result (Ref. Range & Units)
Lupus Anticoagulant Test	Negative
Antinuclear Antibodies, Immunofluorescent Assay	Negative
Double-Stranded Deoxyribonucleic Acid	5 (1-4 IU/mL)
Chromatin Antibody	Negative
Ribosomal P Antibody	Negative
Smith Antibody	Negative
Small Nuclear Ribonucleic Protein Antibody	Negative
Ribonucleic Protein Antibody	Negative
Beta-2 Antibody	Negative
Cardiolipin Antibody	Negative
Beta-2 Glycoprotein 1 Antibody	Negative
Cardiolipin Immunoglobulin G Antibody	Negative
Beta-2 Immunoglobulin M Antibody	Negative
Cardiolipin Immunoglobulin M Antibody	Negative
Beta globulin, Urine	20.2 [%]
Immunoglobulin G, Cerebrospinal Fluid	2.6 [<=8.1 mg/dL]
Immunoglobulin G Index, Cerebrospinal Fluid	0.50 [<=0.85]
Immunoglobulin G/Albumin, Cerebrospinal Fluid	0.16 [<=0.21]
Oligoclonal bands, Cerebrospinal Fluid	0
Immunoglobulin G, Serum	1370 [767-1590 mg/dL]
Gamma globulin, Urine	15.7 [%]
Oligoclonal Bands, Serum	0
Sjögren's Syndrome A Antibody	Negative
Sjögren's Syndrome B Antibody	Negative
Anti-Scleroderma-70 Antibody	Negative
Anti-Jo-1 Antibody	Negative
Centromere Antibody	Negative
Anti-Centromere B Antibody	<0.2 [0.0-0.9 Al]
Anti-Myeloperoxidase Antibody	<9.0 [0.0-9.0 U/mL]
Anti-Proteinase 3 Antibodies	<3.5 [0.0-3.5 U/mL]
Atypical Perinuclear Anti-Neutrophil Cytoplasmic Antibodies	Negative
Cyclic Citrullinated Peptide Antibodies Immunoglobulin G/ Immunoglobulin A	5 [0-19 units]
Rheumatoid Arthritis Latex Turbid	<10.0 [0.0-13.9 IU/mL]
Antinuclear Antibody HEp-2 Substrate	Positive 1:160
Antinuclear Antibody HEp-2 Titer	1:80
Antinuclear Antibody HEp-2 Pattern	Homogeneous
Antinuclear Antibody HEp-2 Pattern (2)	Nucleolar
Angiotensin-Converting Enzyme Level	34 U/L [14-82 U/L]
Alpha-1-Globulin	0.4 [0.0-0.4 g/dL]
Alpha-1-Globulin, Urine	5.4 [%]
Alpha-2-Globulin	1.0 [0.4-1.0 g/dL]
Alpha-2-Globulin, Urine	27.5 [%]
Immunoglobulin G, Subclass 1	1,239 [248-810 mg/dL]
Immunoglobulin G, Subclass 2	399 [130-555 mg/dL]
Immunoglobulin G, Subclass 3	56 [15-102 mg/dL]
Immunoglobulin G, Subclass 4	60 [2-96 mg/dL]
Beta Globulin	1.4 [0.7-1.3 g/dL]
Monoclonal-Spike	Not observed
Neuromyelitis Optica Immunoglobulin G Autoantibodies	<1.5 [0.0-3.0 U/mL]
Infectious Workup	
Glucose, Cerebrospinal Fluid	68 [40-70 mg/dL]
Protein, Cerebrospinal Fluid	285 [150-450 mg/L]
Albumin, Cerebrospinal Fluid	16.3 [<= 27.0 mg/dL]
Coccidioidomycosis Antibody, Immunoglobulin G, Cerebrospinal Fluid	Negative
Coccidioidomycosis Antibody, Immunoglobulin M, Cerebrospinal Fluid	Negative
Coccidioidomycosis Antibody, Cerebrospinal Fluid	Negative
Coccidioides Antibody Immunoglobulin G, Enzyme-Linked Immunosorbent Assay	<0.150 [Abs]
Coccidioides Antibody Immunoglobulin M, Enzyme-Linked Immunosorbent Assay	<0.150 [Abs]
Hepatitis B Core Antibody, Total	Negative
Hepatitis B Surface Antigen Screen	Negative
Hepatitis B Surface Antibody, Qualitative	Reactive
Hepatitis B Surface Antibody Quantitative	13.1 [Immunity>9.9 mIU/mL]
Hepatitis C Virus Antibody	Negative
Adenovirus	Not Detected
Metapneumovirus	Not Detected
Rhinovirus/Enterovirus	Not Detected
Influenza A	Not Detected
Influenza A, H1 Subtype	Not Detected
Influenza A, H3 Subtype	Not Detected
Influenza A H12009	Not Detected
Influenza B	Not Detected
Parainfluenza 1	Not Detected
Parainfluenza 2	Not Detected
Parainfluenza 3	Not Detected
Parainfluenza 4	Not Detected
Respiratory Syncytial Virus	Not Detected
Bordetella Parapertussis, Respiratory Panel	Not Detected
Bordetella Pertussis, Respiratory Panel	Not Detected
Chlamydia Pneumoniae, Respiratory Panel	Not Detected
Mycoplasma Pneumoniae, Respiratory Panel	Not Detected
Coronavirus HKU1	Not Detected
Coronavirus NL63	Not Detected
Coronavirus 229E	Not Detected
Coronavirus OC43	Not Detected
Coronavirus SARS CoV 2	Not Detected
Chlamydia Trachomatis Deoxyribonucleic Acid	Not Detected
SARS-CoV-2 Ribonucleic Acid	Negative
Neisseria Gonorrhea Deoxyribonucleic Acid	Not Detected
Rapid Plasma Reagin	Non-Reactive
Venereal Disease Research Laboratory, Spinal Fluid	Non-Reactive
Bartonella Henselae Immunoglobulin G	Negative
Bartonella Henselae Immunoglobulin M	Negative
Bartonella Quintana Immunoglobulin G	Negative
Bartonella Quintana Immunoglobulin M	Negative
Brucella Antibody Immunoglobulin G, Enzyme-Linked Immunosorbent Assay	Negative
Cysticercosis Antibody, Immunoglobulin G	0.2 [<=0.8 IV]
Helicobacter Pylori Stool Antigen, Enzyme-Linked Immunosorbent Assay	Negative
Helicobacter Pylori, Immunoglobulin G Antibodies	0.21 [0.00-0.79 Index Value]
Histoplasma Mycelial Antibody	Negative
Histoplasma Yeast Antibody	Negative
Human Immunodeficiency Virus Screen Fourth Generation With Reflex	Non-Reactive
Q Fever Phase I	Negative
Q Fever Phase II	Negative
QuantiFERON Tuberculosis Antigen	0.04 [IU/mL]
Toxoplasma Gondii Antibody, Immunoglobulin G	<3.0 [0.0-7.1 IU/mL]
Toxoplasma Gondii Antibody, Immunoglobulin M	<3.0 [0.0-7.9 AU/mL]
Malignancy Workup	
Carcinoembryonic Antigen	2.0 [0.0-4.7 ng/mL]
Cancer Antigen-125	12.3 [0.0-38.1 U/mL]
CD45	Immunophenotyping by flow cytometry of cells from inguinal lymph node tissue shows no abnormal T cells or monotypic B cells.
CD2	
CD3	
CD4	
CD5	
CD7	
CD8	
CD10	
CD19	
CD20	
Kappa, surface	
Lambda, surface	
Miscellaneous	
Lead, Blood	<2 [0-4 kg/dL]
Calcium	9.7 [mg/dL]

While on prednisone, she underwent EGDs and a colonoscopy showing benign gastric and colonic polyps. She was also diagnosed with right ovarian vein thrombosis and underwent anticoagulation therapy with apixaban for three months, which resolved. After trialing dicyclomine, nortriptyline, pantoprazole, and ursodiol, she found no improvement in gastrointestinal symptoms including abdominal pain, nausea, constipation, and diarrhea. Over the next nine months, repeat MRIs showed lesions in the cervical spine, left frontal lobe, and left optic nerve, again concerning for malignancy. She was referred to ophthalmology, infectious disease, and neurosurgery. Vascular surgery diagnosed her right labial swelling as varicosities. Repeated CT and PET scans demonstrated new areas of LAD; however, there was resolution of the mediastinal LAD and pulmonary nodules. Her temporal artery biopsy and lumbar puncture were negative, and brain biopsy was not done given the high-risk nature. There was suspicion of Castleman disease, but additional workup was negative except for elevated IgG1 and positive antinuclear antibody Hep-2 substrate, which was thought to be non-specific and related to her inflammatory arthritis, respectively (Table 1). She then started a four-week course methylprednisolone taper at 48 mg daily for optic nerve involvement.

Upon additional imaging in the following year, her mediastinal LAD reappeared prompting an endobronchial ultrasound bronchoscopy with transbronchial needle aspiration revealing a non-caseating granuloma. This clenched the diagnosis of sarcoidosis with presumption of neurosarcoidosis, given brain lesions. During the next seven months, she was trialed on methotrexate 15 mg once a week for three months but stopped due to gastrointestinal intolerance. She was then started on infliximab at 400 mg for 15 weeks but stopped because of generalized swelling; she still had persistent optic nerve lesions. She took mycophenolate at 1000 mg twice a day for two months but also stopped due to intolerance. Her current treatment is adalimumab 40 mg injection every two weeks. Repeat imaging of her brain and chest showed resolving LAD, and she had improvement in headaches with no fevers but still had persistent fatigue. She is regularly following up with rheumatology, internal medicine, and her other specialists with plans for repeat laboratory tests and imaging.

## Discussion

The incidence rate of sarcoidosis varies from 2.3 to 17.8 for every 100,000 people per year in different cohorts of patients in different regions, making it a very rare disease [3,4]. Moreover, sarcoidosis has a variable presentation often mimicking other conditions. It is a multisystemic disease with a wide spectrum of symptoms including fatigue, memory loss, dyspnea, cough, pain, and dizziness [5]. Additionally, diagnostic tests are often suggestive rather than pathognomonic. Although there are many cases in the English literature reflecting difficult diagnosis, this case illustrates a unique and challenging presentation of sarcoidosis because of chronic, fluctuating, diffuse LAD with a nonresponse to steroids [6-8]. Prognosis of the disease often relies on a variety of factors including organ involvement, response to medications, race, and age [9]. Particularly with neurosarcoidosis, there is no clear prognosis; however, some studies suggest response to treatment improves outcomes [10]. 

There is no diagnostic test for sarcoidosis; multiple criteria need to be met, including clinical and imaging evidence, non-caseating granuloma on biopsy, and exclusion of other diseases [11]. Workup tends to be considerable and time-consuming, leading to a delayed diagnosis while pursuing multiple empiric treatments as experienced by our patient. Her initial FNA biopsy of the submandibular gland was thought to be non-specific in the setting of negative angiotensin-1 converting enzyme levels, which are reported to be elevated in 75% of sarcoidosis patients, swaying sarcoidosis away from diagnosis initially [11]. In addition, multiple attempts at biopsies, including excisional biopsies, were unsuccessful due to lack of appropriate targets. Differential diagnoses were broad and consisted of IgG4 disease, neurocysticercosis, tuberculosis, giant cell arteritis, Castleman disease, lymphoma, and others. Clenching the diagnosis of sarcoidosis often requires serendipity, as in our case. It was only after multiple biopsies that revealed one non-caseating granuloma. Moreover, the patient’s nonresponse to corticosteroids, a cornerstone treatment of sarcoidosis, brought uncertainty. The fluctuating nature of her condition hindered early diagnosis, which is crucial in suppressing inflammatory and immunological processes, and made treatment challenging at times [12]. Multiple medications were ineffective; however, adalimumab injection yielded positive results.

## Conclusions

We presented a rare case of a patient with chronic, fluctuating diffuse lymphadenopathy, escaping sarcoidosis diagnosis for eight years despite substantial workup. She failed multiple antibiotic treatments and various imaging showed widespread lymphadenopathy, stimulating concern for malignancy. It was only after multiple biopsies that one non-caseating granuloma was revealed. However, uncertainty still loomed as the patient was initially non-responsive to corticosteroids, a cornerstone treatment of sarcoidosis. There should be a high index of suspicion for a rheumatologic process like sarcoidosis for patients with similar history and presentation.
